# Transitioning of Registered General Nurses to Nurse Managers—A Quantitative Study in the Greater Accra Region of Ghana

**DOI:** 10.1155/jonm/7932252

**Published:** 2026-02-03

**Authors:** Docia Baah, Theresa Barnes, Adelaide Maria Ansah Ofei

**Affiliations:** ^1^ Department of Research, Education and Administration, School of Nursing and Midwifery, University of Ghana, Legon, Ghana, ug.edu.gh

## Abstract

**Background:**

Globally, transitioning is inevitably marked by shifts from one phase to another, reflecting varied life changes. These transitions, identified in diverse studies, encompass positive, negative, or nonevent occurrences and profoundly influence individuals’ psychological processes as they adapt to external changes. Using Schlossberg’s transition theory as the organizing framework, the transitioning of registered general nurses (RGNs) to nurse managers (NMs) in the Greater Accra Region of Ghana was studied.

**Method:**

A descriptive cross‐sectional survey design was used to examine the transitioning of NMs. Multistage sampling was utilized to select 103 NMs from the Greater Accra Region of Ghana.

**Results:**

NMs moving through transition was efficient to a large extent with a mean score of 3.64 (SD = 0.54), the overall mean score obtained for moving out (taking charge) of transitions was 4.07 (SD = 0.68). Moving through was related to moving out (*r* = 0.29, *p* < 0.05), strategies (*r* = 0.54, *p* < 0.001), support (*r* = 0.52, *p* < 0.001), self (*r* = 0.77, *p* < 0.001), and situation (*r* = 0.65, *p* < 0.001). Finally, moving out was found to be positively and significantly related to three other scores: strategies (*r* = 0.41, *p* < 0.001), support (*r* = 0.52, *p* < 0.001), and self (*r* = 0.77, *p* < 0.001).

**Conclusion:**

The study concluded that NMs received support in this current study, which is in contrast to other studies. RGNs faced certain challenges in transitioning to NMs primarily related to planning, but they demonstrated high degrees of optimism and control. Additionally, actively “taking charge” of the transition is successful when strategies such as seeking advice and negotiation are employed.

## 1. Introduction

Transitioning marks the conclusion of one life phase and the commencement of a new one, requiring the development of new perspectives and adaptive strategies to meet evolving demands [1]. Characterized by moments of joy, uncertainty, and sometimes shock, this process typically involves individuals in unlearning and relearning to integrate into their new environment successfully. This phenomenon applies to NMs in transition [2].

The frequent turnover of registered general nurses (RGNs) to nurse manager (NM) positions poses a significant challenge, particularly in high‐income countries, where aging nursing workforces and elevated attrition rates exacerbate the issue [3]. Insufficient institutional support and elevated stress levels have contributed to a diminished interest in nurse managerial roles, with fewer than 12.5% of nurses in the United States expressing a willingness to pursue this career path [4].

Similarly, in low‐income countries, RGNs are frequently promoted to NM roles at the ward level due to staff transfers, retirements, and emigration to higher‐income countries [5]. In Ghana, recent trends in international nurse migration have intensified this dynamic, leading to the departure of experienced nursing personnel from the health sector [6]. Ensuring the continuity of effective ward‐level management necessitates the timely replacement of these NMs to sustain operational efficiency within the Ghanaian healthcare system.

The NM plays a critical role in promoting the effective delivery of care to patients and families while also supporting staff performance and well‐being within the ward environment [7]. However, the transition to a leadership role is often abrupt and challenging, offering limited time for adjustment. This can result in negative consequences, including reduced patient satisfaction, low staff morale, increased turnover, and a diminished professional climate [8].

Achieving role mastery is vital after such transitions, with the concept of “moving out” (taking charge) representing the acquisition of additional responsibilities NMs must assume. Two nursing theories, Kramer’s reality shock theory and Benner’s novice to expert model, are closely connected to Schlossberg’s transitioning theory.

Kramer’s reality shock theory aligns with Schlossberg’s “situation” in transition. The interplay between administrative ideals and operational realities can lead to shock among NMs, initially causing stress and disappointment and making the challenges they face tangible [9].

Additionally, Benner’s novice to expert model according to Benner [10] highlights the “self” and “strategies” components of Schlossberg’s theory. The “self,” which looks at the NMs personal characteristics as leaders, is likened to Benner’s clinical expert to an administrative novice. Thus, the NM, who is a clinical expert, becomes an administrative novice. Consequently, transitioning provides NMs with an avenue to draw on “strategies” to “move out” successfully.

This study aimed to assess the coping resources utilized by NMs during their transition in the Greater Accra Region of Ghana and to examine the “moving out” (taking charge) phase of the transition process. Although studies have explored NM transitions, there is a significant gap in quantitative research examining the transitioning of RGNs to NM. To address the methodological gap, the researchers sought to measure relationships among variables such as moving through (situation, self, support, and strategies) and moving out (taking charge) during transitions among NMs.

## 2. Methods

### 2.1. Design

A descriptive cross‐sectional survey design was employed for this study.

### 2.2. Setting

The research was conducted among NMs working in public health facilities at the Greater Accra Region of Ghana’s primary, secondary, and tertiary levels. Description of the various levels of hospitals according to the Ghana Statistical Service and the Ministry of Health, Ghana [11], and WHO [12] is as follows.

#### 2.2.1. Primary/District Hospital

The district health management teams oversee primary hospitals. District hospitals are facilities for clinical care at the district level. The beds in district hospitals typically range from 50 to 60. They act as the first referral hospitals. These hospitals provide twenty‐four‐hour services, including curative, preventive, and health promotion activities for the people in the district. Inpatient care is also offered until the patient can either go home or return to the health center.

#### 2.2.2. Secondary/Regional Hospital

The Regional Health Directorate oversees this level. They provide services to a clearly defined geographical area with a population of about 1.2 million. Regional hospitals generally have a capacity of 150–200 beds. They offer specialized care such as medicine, general surgery, anesthesia, pediatrics, obstetrics and gynecology, dental services, psychiatry, ear, nose, and throat, ophthalmology, dermatology, and other services not available at the primary level, making them the next step in referral.

#### 2.2.3. Tertiary/Teaching Hospital

Governance of Teaching Hospitals is unusual because it involves many players, such as the Ministry of Health, the Ministry of Education, Universities, and Political influences in the community. Teaching hospitals are centers of excellence and complex health care. The care at these facilities requires more complex technology and highly skilled personnel. They have a high concentration of resources and are relatively expensive to run. They also support the training of health workers with both preservice and in‐service teaching as well as providing solutions to health problems through research. As the nation’s capital, the Greater Accra Region is the smallest by land area but the second most populous among Ghana’s sixteen regions.

### 2.3. Sample

A multistage sampling strategy was employed. First, eight hospitals were selected using simple random sampling. This was followed by a census‐based sampling method to select respondents. Due to the limited and dispersed population of NMs within the eight hospitals, a census approach was adopted for respondent recruitment. The census method maximized participation, which was essential to the quantitative study’s statistical power and generalizability [13].

All eligible NMs who provided informed consent were included in the study sample. There were 103 respondents in all. Eligibility criteria took into consideration NMs working in government hospitals with more than 6 months of work experience and who had direct nursing care responsibilities toward patients.

Data were collected using the Transition Guide & Questionnaire (TGQ), developed by Schlossberg and Kay [14], which was designed to assess transitioning among professionals. Sample distribution across the level of hospitals is presented in Table 1.

**Table 1:** Levels of facility.

Level of facility	Frequency	Percentage
Primary	22	21.4
Secondary	**39**	**37.9**
Tertiary	**42**	**40.7**
Total	103	100

*Note:* The bold values represent the frequencies and percentages of the facility levels. The values 39, 37.9, 42, and 40.7 indicate that secondary and tertiary facilities are the most prevalent types in this data set, suggesting that the study’s focus is primarily on these higher levels of care delivery.

### 2.4. Instrumentation

#### 2.4.1. Validity and Reliability

Validity and reliability were ensured as follows: Face and content validity were ensured in this study. Face validity was guaranteed by giving the questionnaire to colleagues, supervisors, and friends to see whether the expected responses will be elicited. In addition, pretest of the tool was done using 30 respondents. On completion of the pretest by the respondents, the responses gathered were found to be valid.

Additionally, the TGQ had been used in many areas such as humanities, health, and education and had proven to be valid and reliable [15–19]. In an exhaustive literature review, the tool was found to contain sufficient questions to answer the objectives of the study [20]. Additionally, pretest of the tool was done using 10% of the respondents. The pretest was done at the Lekma General Hospital in Teshie, a suburb of Accra. On completion of the pretest by the respondents, the responses gathered were found to be valid.

Reliability concerns the instrument’s ability to produce similar results when tested at different times [21]. It explains that reliability is measured by calculating Cronbach’s alpha coefficient, which ranges from 0.00 to 1.0. The closer the Cronbach’s alpha value is to 1, the more consistent the scales in the instrument are considered [22]. Using Statistical Package for Social Sciences (SPSS), the overall Cronbach’s alpha was calculated to be 0.893, indicating strong reliability of the tool used. A breakdown of Cronbach’s alpha values for the scales is presented in Table 2.

**Table 2:** Cronbach values.

Cronbach values (scale)	Reliability
Approaching transition	0.712
Situation	**0.954**
Self	0.887
Support	0.676
Strategies	**0.954**
Outcome	**0.967**

*Note:* Two structured questionnaires were used for data collection. Part A consisted of *the Transition Guide & Questionnaire (TGQ)*, originally developed by Schlossberg and Kay (2003), while Part B captured data on “taking charge” during the transition phase, based on role mastery indicators proposed by the authors in [23]. The TGQ was organized into four subscales. The bold values indicate a high level of internal consistency reliability for the respective scales. The Cronbach’s alpha values for situation, strategies, and outcome (0.954, 0.954, and 0.967, respectively) are all above the commonly accepted threshold of 0.7 and, specifically, above 0.9, suggesting excellent reliability. which means the items within each of these scales consistently measure the same underlying construct, making the scales highly dependable and stable for the research purpose.

##### 2.4.1.1. Situation Subscale

This section included 10 items rated on a 5‐point Likert scale, where 1 represented a negative response and 5 a positive one. Two items that assessed stress during the transition period were reverse‐coded, with 1 indicating *high stress* and 5 indicating *low stress*. For clarity and contextual relevance, the term “situation” in the original tool was replaced with “being a Nurse Manager.”

##### 2.4.1.2. Self‐Subscale

This included 10 items measuring aspects such as sense of control, optimism/pessimism, resilience, self‐knowledge, emotional energy, personal values, self‐blame, self‐perception, and the extent to which personal needs and expectations were met during the transition. Nine of the items were rated on a 5‐point Likert scale ranging from 1 = “*Not at all*” to 5 = “*To a very large extent*.” One item was reverse‐coded and retained in its original format.

##### 2.4.1.3. Support Subscale

Initially consisting of nine items, one item—*“I have a variety of support activities (intellectual pursuits, hobbies, athletics, and volunteerism)”*—was removed to enhance contextual appropriateness. Consequently, removing the item did not affect the tool’s overall Cronbach alpha value. The remaining eight items assessed perceived support from family, friends, and significant others. Responses were rated on a 5‐point Likert scale: 1 = “*Never*,” 2 = “*Rarely*,” 3 = “*Occasionally*,” 4 = “*Often*,” and 5 = “*Always*.”

##### 2.4.1.4. “Moving Through” Strategies Subscale

This subscale comprised 27 items measuring the effectiveness of coping and adaptation strategies employed by NMs during their transition. A 5‐point Likert scale was used, with 1 = “*Very ineffective*” and 5 = “*Very effective*.”

Part B measured outcomes related to the process of “taking charge” during the transition, aligned with the *Moving Out* phase of Schlossberg’s transition theory. It contained four subscales, each assessed on a 5‐point Likert scale where 1 = “*Not at all*” and 5 = “*To a very large extent*.” The items were adapted from Warshawsky and Cramer [23] framework on NM role mastery to evaluate successful transition outcomes among NMs.

### 2.5. Ethical Consideration and Data Collection

Ethical approval for the study was obtained from the Ghana Health Service Ethics Review Committee (GHSERC), with study number GHS: 066/05/23. Data collection was carried out between 30th July and 15th September 2023. All respondents provided informed consent prior to their inclusion in the study. Confidentiality and anonymity were maintained throughout the research process.

### 2.6. Data Analysis

Data were analyzed using the SPSS Version 23.0. The analysis followed the scoring and interpretation guidelines outlined in the TGQ developed by Schlossberg and Kay (2003). Descriptive statistics, including frequencies, means, standard deviations, and percentages, were used to summarize respondents’ sociodemographic characteristics.

To examine relationships between the subscales, Spearman’s rho correlation was employed to test the association between the *Moving Through* and *Moving Out* variables.

## 3. Results

Sociodemographic and professional characteristics of respondents are presented in Table 3. Most of the respondents were predominantly female, (*n* = 76, 73.8%), aged 30–44 years (*n* = 35, 34%), and held first degree (*n* = 56, 54.4%) and SNO (*n* = 35.9%). Respondents had largely worked for 5–9 years RGNs (*n* = 40, 38.8%) before being assigned the NM’s role.

**Table 3:** Sociodemographic and professional characteristics of NMs (*n* = 103).

Variables	Categories	Frequency	Percentage (%)
Age (years)	30–34	**35**	**34.00**
	35–39	28	27.20
	40–44	32	31.10
	≥ 45	8	7.80

Gender	Males	27	26.20
	Females	**76**	**73.80**

Rank	DCNOs	16	15.50
	PNO	**39**	**37.90**
	SNO	37	35.90
	NO	11	10.70

Highest qualification	Diploma	6	5.80
	First degree	**56**	**54.40**
	Master’s	41	39.80

*Note:* Source: Field Data, 2023. The bold values represent the highest frequency and percentage within each variable category. For age (years), the bold frequency is 35, and the percentage is 34.00%, the bold frequency is 76, and the percentage is 73.80%, for rank, the bold frequency is 39 and percentage is 37.90% and for highest qualification, the bold frequency is 56, and the percentage is 54.40%.

### 3.1. Situation of NMs—How Do You See the Transition

In Table 4, the overall mean score is 3.29 (SD = 1.00), which describes the situation of NMs in moving through the transitional experience.

**Table 4:** Situation of NMs—How do you see the transition?

Situation of NMs	1 (%)	2 (%)	3 (%)	4 (%)	5 (%)	M (SD)(%)
How do you see the transition? Looking ahead I feel able to plan with difficulty.	10 (9.7)	12 (11.7)	36 (35)	34 (33)	11 (10.7)	**3.29 (1.01)** 3.23 (1.10)
For me, being a nurse manager is happening at the best possible moment.	23 (22.3)	16 (15.5)	16 (15.5)	26 (25.2)	22 (21.4)	3.08 (1.47)
I see being a nurse manager as being within personal control.	24 (23.3)	10 (9.7)	14 (13.6)	24 (23.3)	31 (30.1)	3.27 (1.55)
I view this situation as desirable.	18 (17.5)	19 (18.4)	24 (23.3)	19 (18.4)	23 (22.3)	3.10 (1.40)
From where I stand now, the nurse manager role is likely to be of manageable duration.	17 (16.5)	16 (15.5)	12 (11.7)	24 (23.3)	34 (33)	3.41 (1.49)
The outcome of the transition is likely to be positive.	15 (14.6)	8 (7.8)	12 (11.7)	22 (21.4)	46 (44.7)	3.74 (1.46)
I expect to bring to this transition the great benefits of previous experience.	15 (14.6)	7 (6.8)	17 (16.5)	23 (22.3)	41 (39.8)	3.66 (1.43)
In my culture, this transition would easily be accepted.	23 (22.3)	8 (7.8)	10 (9.7)	30 (29.1)	32 (31.1)	3.39 (1.54)
I am currently dealing with other concurrent stressors in my life.	17 (16.5)	12 (11.7)	20 (19.4)	34 (33)	20 (19.4)	3.27 (1.35)
This transition will be likely to cause much stress in other roles in my life.	23 (22.3)	22 (21.4)	22 (21.4)	20 (19.4)	16 (15.5)	2.84 (1.38)

*Note:* Key: 1 = not at all, 2 = to a small extent, 3 = to some extent, 4 = to a large extent, and 5 = to a very large extent. M = mean; source: Field Data, 2023. The bolded values in the table represent the mean (M) score for each survey statement. These values indicate the average extent to which the nurse managers agreed with the situation described in each statement, based on a 5‐point Likert scale provided in the note below the table.

Abbreviation: SD = standard deviation.

### 3.2. Self of NMs—Who Are You

Table 5 presents the scores rated by the NMs on self which describes who they are in facing transitions. The cumulative mean score and standard deviation on this item was 3.81 (0.67).

**Table 5:** Self of NMs—Who are you?

Self of NMs	1 (%)	2 (%)	3 (%)	4 (%)	5 (%)	M (SD)
**Who are you?** I feel a sense of control or mastery as I face transitions.	8 (7.8)	16 (15.5)	16 (15.5)	36 (35)	27 (26.2)	**3.81 (0.67)** 3.53 (1.25)
I usually face life as an optimist.	9 (8.7)	2 (1.9)	12 (11.7)	32 (31.1)	48 (46.6)	4.05 (1.21)
When I think about how resilient I am in the face of change, I would describe myself as extremely resilient.	7 (6.8)	2 (1.9)	20 (19.4)	36 (35)	38 (36.9)	3.93 (1.12)
I feel that I know myself.	7 (6.8)	2 (1.9)	14 (13.6)	36 (35)	44 (42.7)	4.05 (1.12)
In responding to this transition, I find myself with ample physical and emotional strength.	20 (19.4)	8 (7.8)	16 (15.5)	37 (35.9)	32 (21.4)	3.32 (1.41)
This transition is in line with my values.	12 (11.7)	6 (5.8)	9 (8.7)	43 (41.7)	33 (32)	3.77 (1.29)
I feel good about myself.	5 (4.9)	4 (3.9)	15 (14.6)	30 (29.1)	49 (47.6)	4.11 (1.10)
I know how to meet my needs when going through transitions.	3 (2.9)	2 (1.9)	12 (11.7)	54 (52.4)	32 (31.1)	4.07 (0.88)
My expectations are realistic.	2 (1.9)	8 (7.8)	58 (56.3)	35 (34)	—	4.20 (0.75)
When things go wrong, I blame myself.	20 (19.4)	9 (8.7)	34 (33)	20 (19.4)	20 (19.4)	3.11 (1.36)

*Note:* Key: 1 = never, 2 = rarely, 3 = occasionally, 4 = often, and 5 = always. M, mean; source: Field Data, 2023. The bold values are the mean (M) scores and standard deviation (SD) for each survey item, indicating the average response on a scale of 1 (never) to 5 (Always). The ‘M’ values provide a single, central figure summarising the typical response for each statement, allowing for comparison of average sentiment across different items.

Abbreviation: SD = standard deviation.

### 3.3. Support of NMs From Significant Others

In Table 6, NMs’ support received during transition obtained a mean score of 3.73 (SD = 0.77).

**Table 6:** Support of NMs from significant others.

Support of NMs	1 (%)	2 (%)	3 (%)	4 (%)	5 (%)	M (SD)
**What help you have from others** My family provides fully adequate support	8 (7.8)	2 (1.9)	20 (19.4)	34 (33)	39 (37.9)	**3.73 (0.77)** 3.91 (1.16)
My spouse or partner provides fully adequate support	24 (23.3)	4 (3.9)	10 (9.7)	24 (23.3)	41 (39.8)	3.52 (1.60)
My friends provide fully adequate support	8 (7.8)	6 (5.8)	16 (15.5)	50 (48.5)	23 (22.3)	3.72 (1.12)
A group, other than my family or friends (coworkers, support groups, or other professional groups)	6 (5.8)	8 (7.8)	32 (31.1)	38 (36.9)	19 (18.4)	3.54 (1.06)
Affection needs fully met	9 (8.7)	4 (3.9)	21 (20.4)	33 (32)	36 (35)	3.81 (1.21)
Respect for the way I am handling this transition is of a high degree	4 (3.9)	4 (3.9)	14 (13.6)	40 (38.8)	41 (39.8)	4.07 (1.02)
Assistance	12 (11.7)	8 (7.8)	22 (21.4)	30 (29.1)	31 (30.1)	3.58 (1.31)
Much feedback.	4 (3.9)	9 (8.7)	27 (26.2)	35 (34)	28 (27.2)	3.72 (1.08)

*Note:* Key: 1 = never, 2 = rarely, 3 = occasionally, 4 = often, and 5 = always. M, mean; source: Field Data, 2023. The ‘M’ column in the table indicates the mean score for each item, which is a measure of central tendency. The values are bolded to highlight the average level of support reported by the respondents for each specific type of help or need satisfaction. For example, the mean of 4.07 for “Affection needs fully met” indicates a high degree of met needs, as the scale key notes 4 = often and 5 = always.

Abbreviation: SD = standard deviation.

### 3.4. Strategies of NMs—How You Manage Change

The composite and subscales scores rated by the NMs regarding strategies used in managing their changed roles are presented. The findings revealed that the overall mean score for the subscale strategies was 3.74 (SD = 0.67) which suggested that the strategies used in transition were effective among NMs in the Greater Accra Region of Ghana.

### 3.5. Moving out (Taking Charge) of Transitioning Among NMs

Table 7 presents the overall mean score obtained for moving out (taking charge) of transitions to be 4.07 (SD = 0.68), which depicts that to a large extent, taking charge is effective among nurses who transition to the NM role.

**Table 7:** Strategies of NMs—How you manage change?

Strategies of NMs	1 (%)	2 (%)	3 (%)	4 (%)	5 (%)	M (SD)
**How you manage change** Negotiating (compromising, talking things through).	6 (5.8)	2 (1.9)	11 (10.7)	44 (42.7)	40 (38.8)	**3.74 (0.67)** 4.07 (1.05)
Taking action (mobilizing yourself and your resources, making a plan and carrying it out).	9 (8.7)	2 (1.9)	15 (14.6)	41 (39.8)	36 (35)	3.90 (1.17)
Seeking advice (through books or asking others for guidance).	6 (5.8)	4 (3.9)	10 (9.7)	31 (30.1)	52 (50.5)	4.16 (1.13)
Asserting yourself (standing up for yourself).	7 (6.8)	—	12 (11.7)	24 (23.3)	60 (58.3)	4.26 (1.12)
Applying knowledge of the transition process (recognizing that all change requires adaptation and time to adjust).	3 (2/9)	1 (1)	7 (6.8)	34 (33)	58 (56.3)	**4.93 (0.89)**
Balancing your work, family, and leisure roles (able to devote time to all aspects of yours).	12 (11.7)	2 (1.9)	15 (14.6)	38 (36.9)	36 (35)	3.82 (1.27)
Playing (allowing the child within to emerge and have fun).	5 (4.9)	9 (8.7)	24 (23.3)	41 (39.8)	24 (23.3)	3.68 (1.08)
Using relation skills (controlling physical reactions to stressful situations through relaxation tapes, biofeedback, muscle relaxation, and/or visualization).	8 (7.8)	1 (1)	20 (19.4)	53 (51.5)	21 (20.4)	3.78 (1.01)
Expressing emotions (letting off steam through crying, yelling, or vigorous physical activity).	14 (13.6)	12 (11.7)	29 (28.2)	25 (24.3)	23 (22.3)	3.30 (1.31)

*Note:* M, mean; source: Field Data, 2023. The mean scores provide a summary measure of central tendency for each item, allowing for comparison of how frequently or significantly each “how you manage change” strategy is perceived or utilised. For example, “Applying knowledge of the transition process” has a mean score of 4.93, which appears to be the highest among the listed strategies, suggesting it is considered the most significant or most frequently used strategy by the survey respondents. The standard deviation (SD) values, which are in parentheses next to the mean, indicate the variability or spread of the responses around the mean.

Abbreviation: SD = standard deviation.

### 3.6. Relationship Between Moving Through (Situation, Self, Support, and Strategies) and Moving out (Taking Charge) of NMs

Table 8 presents the inferential analysis between the variables moving through (situation, self, support, and strategies) and moving out. Spearman’s rho correlation was used to determine the association between the variables moving through (taking charge) and moving out. Thus, overall moving through was related to moving out (*r* = 0.29, *p* < 0.05), strategies (*r* = 0.54, *p* < 0.001), support (*r* = 0.52, *p* < 0.001), self (*r* = 0.77, *p* < 0.001), and situation (*r* = 0.65, *p* = 0.001). Similarly, moving out was found to be positively and significantly related to three other scores. These scores were strategies (*r* = 0.41, *p* < 0.001), support (*r* = 0.52, *p* < 0.001), and self (*r* = 0.77, *p* < 0.001). However, a positive but insignificant association was found between moving out and the situation (*r* = 0.12. *p* = 0.21).

**Table 8:** Strategies of NMs—How you manage change (continued)?

Strategies of NMs	1 (%)	2 (%)	3 (%)	4 (%)	5 (%)	M (SD)
Engaging in physical activity (walking, running, tennis, or exercise of any kind).	9 (8.7)	7 (6.8)	6 (5.8)	45 (43.7)	36 (35)	3.89 (1.21)
Using a range of strategies (knowing there is not one magic strategy but several that may help you manage the transition).	13 (12.6)	5 (4.9)	27 (26.2)	37 (35.9)	21 (20.4)	**3.47 (1.24)**
Brainstorming a new plan (generating all possible suggestions or solutions).	6 (5.8)	—	22 (21.4)	42 (40.8)	33 (32)	3.93 (1.03)
Engaging in humor (improving your laugh life).	9 (8.7)	7 (6.8)	15 (14.6)	32 (31.1)	40 (38.8)	3.84 (1.26)
Having faith (reflecting through prayer, meditation, or solitude).	11 (10.7)	6 (5.8)	4 (3.9)	24 (23.3)	58 (56.3)	4.09 (1.34)
Positive self‐talk (confirming your belief in yourself through verbal affirmations).	9 (8.7)	1 (1)	18 (17.5)	30 (29.1)	45 (43.7)	3.98 (1.20)
Imagine the desired outcome (seeing yourself where you want to be).	14 (13.6)	—	17 (16.5)	32 (31.1)	40 (38.8)	3.82 (1.33)
Being mindful (able to focus on what needs to be done).	4 (3.9)	7 (6.8)	7 (6.8)	25 (24.3)	60 (58.3)	**4.25 (1.10)**

Note: Key: 1 = never, 2 = rarely, 3 = occasionally, 4 = often, and 5 = always. M, mean; source: Field Data, 2023. The table uses a Likert scale where 1 = never, 2 = rarely, 3 = occasionally, 4 = often, and 5 = always. The mean values provide a central tendency measure, showing how often NMs, on average, employ each strategy. For example, a mean of 4.25 for “Being mindful” suggests this strategy is used “often” by NMs, while a mean of 3.47 for “Using a range of strategies” suggests it is used between “occasionally” and “often”. The standard deviation (SD) values, which are in parentheses next to the means, indicate the variability or spread of the responses around the mean.

SD = standard deviation.

Additionally, strategies showed a positive and significant relationship with support (*r* = 0.43, *p* < 0.001) and self (*r* = 0.40, *p* < 0.001) but a positive and insignificant relationship with the situation (*r* = 0.14, *p* = 0.14). Finally, support was positively related to the self (*r* = 0.30, *p* < 0.05) but had a negative and insignificant relationship with the situation (*r* = −0.10, *p* = 0.27) (see​ Tables 9 and 10)

**Table 9:** Moving out of NMs (taking charge).

Items	M	(SD)
**Moving out (taking charge)**	**3.85**	**0.64**
**Changed behavior**	**3.97**	**0.73**
Promote organizational values regarding patient care.	3.87	1.17
Represent the interest of the unit at the departmental and hospital level.	3.99	1.06
Practice what you preach.	3.97	1.08
Work with staff to resolve conflicts with colleagues, physicians, and patient families.	4.05	1.04
**Changed Role**	**4.07**	**0.68**
Ensures that the unit has competent staff needed to provide quality patient care.	3.90	1.05
Make staff nurse performance known and clear.	4.04	1.04
Work together with staff to improve clinical care and unit operations.	4.28	0.86
Ensures that supplies and equipment needed to provide quality patient care are consistently available.	4.09	1.10
**Changed learning**	**3.48**	**0.9**
Guide staff in research and evidence‐based practice activities.	3.27	1.32
Encourage staff to participate in multidisciplinary patient round.	3.68	1.33
Encourage professional development through specialty certification.	3.48	1.24
Solicit staff input in budget and capital expenditure discussions and decisions.	3.49	1.28
**Changed perception**	**3.87**	**0.75**
Provide direct patient care on an ongoing basis.	3.83	1.13
Monitor bed assignment and or follow patients in and out of the clinic.	3.56	1.26
Be involved in day to day direction of unit activities.	3.81	1.04
Provide direct patient care in emergencies or when the unit is usually short of help.	3.93	0.91
Play an active role in the orientation of nurses new to the staff.	4.22	0.89

*Note:* M, mean; source: Field Data, 2003.

Abbreviation: SD = standard deviation.

**Table 10:** Correlation analysis between the variables overall moving through and moving out.

Variable	1	2	3	4	5	6
1	1.000					
	.					

2	0.293^∗∗^	1.000				
	0.003	.				

3	0.547^∗∗^	0.417^∗∗^	1.000			
	0.000	0.000	.			

4	0.520^∗∗^	0.275^∗∗^	0.433^∗∗^	1.000		
	0.000	0.005	0.000	.		

5	0.777^∗∗^	0.195^∗^	0.406^∗∗^	0.303^∗∗^	1.000	
	0.000	0.049	0.000	0.002	.	

6	0.651^∗∗^	0.122	0.144	−0.108	0.495^∗∗^	1.000
	0.000	0.218	0.146	0.276	0.000	.

*Note:* The data indicate a predominantly positive and statistically significant relationship between most variables, with Variable 1 showing the strongest association across the matrix. Nonsignificant results (*p* > 0.05) suggest that certain factors, particularly Variable 6 in relation to Variables 2, 3, and 4, operate independently within this sample.

^∗^Significance at the *p* < 0.05 level.

^∗∗^Significance at the *p* < 0.01 level (2‐tailed).

## 4. Discussion

### 4.1. NMs Moving Through Transition

The situation of RGNs transitioning to NMs in this study was somewhat satisfactory. The high number of NMs suggests that the transition aligns with their values, which can be explained by the fact that the NM position is a reputable one [24]. This may be due to NMs having their own offices in the ward where managerial decisions are made and communicated to the staff, making the power vested in them highly desired by many [25]. It seems that being a NM in some facilities in Greater Accra is sought after by most nurses.

On the other hand, Warden et al. [26] and Labrague [27] have identified a rise in NM’s turnover intentions. Over 50% of NMs in the United States had the intention to leave their current jobs. This is because the NM role is challenging. Chisengantambu et al. [28] highlighted the importance that support plays in achieving organizational goals. In addition to organizational support, other forms of support that were assessed in this study were support from friends, family, and other groups [29].

Additionally, the study found that NMs faced challenges in planning during their transition period. These findings align with earlier research by Ofei and Paarima [6], which reported that NMs showed moderate knowledge of planning, moderate engagement in peer communication, and fair proficiency in planning‐related tasks. Planning tasks usually cover a broad range of areas, including staffing, budgeting, quality improvement initiatives, professional development, interprofessional collaboration, and effective communication, all of which are vital for ensuring optimal patient care outcomes under the leadership of NMs. Managing these diverse responsibilities during a transitional phase can be particularly difficult, often requiring both significant experience and institutional support to facilitate effective role adaptation.

Furthermore, the self‐characteristics that RGNs demonstrated in transitioning to NMs in this current study was often desirable. This was evident in their confidence and resilience. Battistelli et al. [30] agree with the findings that NMs are confident. This group might have developed strategies and experiences or skills that enable them to adapt successfully to transitions. Tau et al. [31] and Boitshwarelo et al. [32] reinforce the resilience inherent in NMs. Their findings emphasized that NMs commonly exhibit moderate to high levels of resilience, a crucial attribute for effectively managing transitions within their roles.

NMs’ resilience among the respondents in this current study may be attributed to the dynamic nature of the healthcare work environment, which involves navigating challenges, supporting teams, adapting to change, balancing priorities, and learning from setbacks [33]. Similarly, NMs’ resilience enables them to provide guidance and support while managing their stressors, thereby ensuring the effective functioning of teams [34]. However, NMs in public hospitals in Botswana were identified as having low resilience.

Contrary to the above, NMs had less confidence, especially in the area of delegation [35]. For instance, Cruz et al. [36] identified optimism to be effective in coping with stress among nurses in Northern Philippines. Whereas optimism was found to be high in male nurses working in public hospitals compared to those in private hospitals. Again, Morsiani et al. [37] assert that leaders who practice inspirational motivation are optimistic about the future and enthusiastic about achieving goals. This current study found a strong sense of control among NMs, which is consistent with global literature that emphasizes the importance of self‐efficacy in successful transitions [38]. Nevertheless, issues of role insufficiency persist [39].

Regarding support, RGNs often had support in transitioning as revealed in this current study. This may be attributed to the fact that NMs in this study influence some decisions that affect their subordinates such as scheduling and appraisal of junior staff which may lead to the support they receive. Many studies that have examined NMs support agree with the findings of this study that the support received by the NMs is varied (Barimani et al. [40], Benner et al. [41], and Hawkley and Kocherginsky [42]).

For instance, Aydogdu [43], in an integrative review, identified numerous studies that found NMs received varied support during the COVID 19 pandemic. Chisengantambu et al. [28] highlighted the importance that support plays in achieving organizational goals. In their study, the sandwich support was used to explain the role of bottom to top and top to bottom support for NMs.

On the contrary, the findings in this current study disagree with other studies, which have indicated that the support received by some NMs is insufficient and have recommended that the management of healthcare institutions should look at the gap and find ways to bridge them (Aydogdu [43], Hewko et al. [44], Penconek et al. [25], and Zwink et al. [24]).

The finding in this current study, which is inconsistent with other studies, may be due to the fact that one of the mandates of Ghana Health Service is to lead interventions which improve the quality of service delivery to its clients. One of the ways to achieve this mandate is through the periodic peer review of health facilities in the country [45]. During this exercise, the regional directorate of the Ghana Health Service deploys senior nurses to assess the various hospitals using a standard checklist. This exercise is accompanied by identification of service delivery gaps, ranking of the health facilities, and commendations.

Consequently, it appears that most hospitals desire to be ranked high and receive commendations from the health directorate. As such, NMs, who are the frontiers of healthcare delivery, are given the needed logistics and support in order to give off their best in the discharge of their duties. The standard deviation of 0.77 derived for the subscale “support” shows that the resopondents’ opinions are close to the mean. It is recommended to the administrative staff, especially, nurse executives, to ensure that NMs are given the needed incentives to reduce the turnover intentions and staff attrition.

Strategies employed by NMs in transitioning were being assertive, applying knowledge of the transition process, and having faith. According to Martins et al. [46], NMs use assertiveness in conflict resolution. Mohammed et al. [47] and Martins et al. [46] agree in their studies that 70% of NMs in Portugal were assertive. On the contrary, an Indonesian study realized that NMs were either passive or aggressive in conflict resolution instead of using assertive communication to address issues [48]. Another study by Ofei [6] showed that NMs in Ghana were more aggressive toward their subordinates (nurses) than other healthcare professional groups.

### 4.2. Moving out (Taking Charge) of Transitioning Among NMs

Four main variables were the focus of moving out of the transitional experience. These were the NMs’ changed behavior, role, learning, and perception. Kodama and Fukahori [49] agree with the findings that NMs’ changed behavior, demonstrated through believing in and showing empathy toward nurses, is highly effective in promoting change. Contrary to the aforementioned, Aldridge [50] suggests that behavioral changes alone might not suffice to ensure effective leadership. A need for a more comprehensive approach, incorporating diverse leadership styles and skills beyond empathy and belief, to foster substantial and lasting changes within nursing environments is important for NMs [51]. Moreover, Heinen et al. [52] affirm that collaborative nursing practice is inherent in some leadership styles.

### 4.3. Relationship Between Moving Through (Situation, Self, Support, and Strategies) and Moving out (Taking Charge)

Spearman’s rho correlation showed moving through was positively and significantly related to moving out. A positive correlation indicates that as “moving through” transitional phases increases, the ability to “move out” of transitions also tends to increase among NMs. The results imply that NMs who exhibited capabilities in situation, self, support, and strategies to a large extent while moving through the transitional phases are better equipped to successfully adjust to their environment which made moving out of transitioning successful [31–33, 53–55]

The findings validate the core tenets of adaptive transition models. Globally, this correlation implies that effective socialization is not passive but active. The dedication required for skill mastery is the central driver for role stabilization (“moving out”).

## 5. Study Limitations

The authors acknowledge the potential for social desirability bias in self‐reported data, where NMs may have inflated responses on valued traits such as resilience. This limitation was controlled by using validated scales and guaranteeing anonymity and confidentiality. While this reduces risk, future research should employ mixed‐methods to triangulate and validate the responses. Although the core stages of transition are universal phenomena, the magnitude of the correlation may vary. Future comparative studies in rural settings are needed to test wider applicability. Notwithstanding these limitations, the result of the study adds to existing knowledge.

## 6. Implication to Nursing Management

The findings of this study show that the better the NM handles the challenges of transition, the faster they settle into their new role. Important practical implications for hospitals and healthcare leaders include the following:

1. Allocating dedicated time and resources to organize a mandatory and structured program tailored at skill building, role clarity, and leadership development capabilities.
2. A policy should be setup for formal mentorship initiatives that pair new NMs with successful leaders.
3. Leadership should implement interventions that reduce stress and role overload for new NMs.

## 7. Conclusion

The study concluded that while RGNs faced moderate planning challenges when transitioning to NM roles, the overall transition was satisfactory. The success was largely attributable to RGNs exhibiting positive traits, receiving support, and employing effective strategies such as assertiveness, applying existing knowledge maintaining faith. In contrast, passive approaches such as inaction or ignoring issues were deemed ineffective. Crucially, taking charge of the transition proved largely successful, supported by a strong correlation indicating that progressing through the transition “moving through” led to a successful completion of the transition “moving out.”

## Ethics Statement

This research was approved by the Ghana Health Service Ethics Review Committee (GHS‐ERC 066/05/23)

## Conflicts of Interest

The authors declare no conflicts of interest.

## Funding

This research was nonfunded.

## Data Availability

The data that support the findings of this study are available on request from the corresponding author, (Theresa Barnes). The data are not publicly available due to restrictions e.g., they containing information that could compromise the privacy of research participants.
